# Second Clinical Report on Continued Fever, Based on an Analysis of Forty-Eight Cases

**Published:** 1851-09

**Authors:** Austin Flint

**Affiliations:** Prof, of the Principles and Practice of Medicine, and Clinical Medicine, in the University of Buffalo


ART. VII.—*Second Clinical Report on Continued Fever, based on an analysis of forty-eight cases.* By AUSTIN FLINT, M. D., Prof. of the Principles and Practice of Medicine, and Clinical Medicine, in the University of Buffalo.

(*Continued from page* 183.)

*Cephalalgia.*— An examination of the histories of the cases with respect to this symptom confirms the several conclusions stated in the first Report. The presence of the symptom was noted in *fourteen* of the twenty-nine cases of *Typhoid;* in *four* of the ten cases of *Typhus*, and in *six* of the nine cases of *Doubtful type;* that is in *twenty-four* or precisely one-half of the forty-eight cases. This is a statement of the proportion of cases in which the symptom existed after the patients came under observation. It is not a fair illustration of the frequency with which the symptom occurs, for, in most of the cases, the patients were not transferred to the hospital at the very beginning of the febrile career. It is to be borne in mind that, in this enumeration, no reference is made to cephalalgia as entering into the access of the disease, but only to its presence after the febrile career is entered upon, in other words, after the time that the patients took to their beds. Had all the patients been under observation from that period, the proportion of cases in which the symptom was present would doubtless have been larger. It is probable that the cases are few in which it is not present, more or less, in the early part of the febrile career. It almost uniformly disappears after the fever has continued for several days. Hence, in many, if not most of the cases in the histories of which it is not noted, it had doubtless existed, and ceased before the patients came under observation. The duration of the symptom, in the cases, respectively, in the histories of which it was noted, was as follows:

*Typhoid.*

| | |
|---|---|
| One day, | Three cases. |
| Two days, | Three cases. |
| Three days, | Three cases. |
| Four days, | Three cases. |
| A few days, | One case. |
| On ninth day, and not previously, | One case. |

*Typhus.*

| | |
|---|---|
| One day, | One case. |
| Four days, | Two cases. |
| Five days, | One case. |

*Doubtful Type.*

| | |
|---|---|
| One day, | One case. |
| Two days, | One case. |
| Three days, | Two cases. |
| Four days, | One case. |
| Few days, | One case. |

In but *three* cases is it noted that the headache returned during the career of the fever. In *one* of these cases it was present in a slight degree from the *seventh* to the *tenth* days inclusive. The disease was mild in this case, and accompanied by spinal tenderness. In *one* case it was present from the *thirteenth* to the *seventeenth* days inclusive, but in this case it occurred in connection with external otitis. In the remaining case it occurred on the *twelfth* day, with an aggravation of symptoms after apparent convalescence.

The symptom was present in *five* of the *seven* fatal cases of *Typhoid*, and in *two* of the *three* fatal cases of *Typhus*.

The diminished acuteness of the perceptive faculty, which is incident to Continued Fever after its continuance for a few days, affords an adequate explanation of the fact that cephalalgia belongs only to the early stage of the disease.

In so far as deductions are admissible from the small number of *Typhus* cases in this collection, it would seem that this symptom is less frequently present after the fever is established, and its duration less than in the *Typhoid* type.

The records of the cases do not state the severity of the cephalalgia, nor whether it was limited to particular portions of the head, save that in a few instances it is noted that the seat of the pain was in the forehead.

*Dizziness*, when the head was raised, was complained of in several cases.

In this, as in the former collection, pains elsewhere than in the head were complained of in a very few cases only. The following are all that the histories contain relative thereto:—In *two Typhoid* cases, on the *third* day after admission, complaint was made of pain in the bowels; in *one* case of the same type, pain in the loins on the *second* day; in *one* case do., pain in arms and feet on the *twelfth* and *thirteenth* days; in *one* case, do., pain in limbs and loins on the *second* day; in *one* case, do., pain in the joints on the *sixth* day; and in *one* case, pain in knee and elbow, with spinal tenderness, on the *fourteenth* day.

In *one Typhus* case, pain in the shoulders and hips was complained of on the *fifth* day; and in *one* case of *Doubtful type*, pain in the abdomen on the *second* day.

The interest in this enumeration lies in the small number of cases in which pains were experienced, the shortness of duration of the pains, and their unimportant character. In this, as in the former collection, painful symptoms were recorded only when mentioned by patients of their own accord, or in answer to a general inquiry as to their condition and feelings. Patients were not asked if they had pain in such and such parts. It is probable that to leading questions the replies might have been frequently in the affirmative, but their correctness could not be relied upon, owing to the mental condition in Continued Fever which has been described. It is reasonable to suppose that the absence of painful sensations in other parts of the body than the head, as well as the cessation of pain in the latter situation, does not denote freedom from morbid conditions ordinarily attended with suffering, but *diminished sensibility.*

As respect lesions of sensibility, the foregoing results are quite different from those developed by the numerical investigations of Louis. In a large proportion of the cases of *Typhoid* fever analyzed by that eminent observer, abdominal pains were more or less present. I can only account for this disparity by supposing that in recording histories, Louis interrogated patients specifically for this symptom. I do not doubt that if this course had been pursued with the patients in this, and the former collection, abdominal, and other pains would have been affirmed in a considerable number of cases. The answers would doubtless have been correct in a certain proportion of cases, for patients with fever may really be conscious of pains, which, from the peculiar condition of mind causing a reluctance to make any effort, they do not complain of, or mention, unless their attention is directed to the subject. The results of my analysis, which relate to *subjective* symptoms, it is to be considered, embrace, for the most part, only the instances in which these symptoms were sufficiently prominent to impel patients to speak of them without addressing leading questions. The reason for this is stated in the preceding paragraph.

*Eye.* The conjunctiva presented increased redness, more or less, in *ten* cases of *Typhoid*, in *three* cases of *Typhus*, and in *four* cases of *Doubtful type*, making, in all, *seventeen* of the *forty-eight* cases.

The proportion of cases in which the presence of this symptom was noted, is somewhat greater than in the first collection, which may be owing to attention having been directed to this point with more care.

Moderate yellowness of the conjunctiva was observed in *two Typhoid* cases.

*Deafness.* Dullness of the sense of hearing sufficient to constitute noticeable deafness, is noted in the histories of *eighteen* of the *forty-eight* cases, viz., in *ten* cases of *Typhoid, five* of *Typhus*, and *three* of *Doubtful type.* It varied in degree, in some cases being considerable, and in other cases slight. It was sometimes the occasion of complaint with patients. When present, it generally made its appearance early, continuing throughout the febrile career, and, occasionally, after convalescence was established.

The proportion of cases in which this symptom was appreciable (as it is not in all cases in which it may be present, for reasons specified in the former Report) differs somewhat, but not greatly in the two collections, the relation being as 18 – 48 is to 24 – 52, or 9 – 24 to 6 – 13. It was present, thus, in *forty-two* of the *one hundred* cases.

In this, as in the first collection, it was present in a larger proportion of the cases of *Typhus*, than in those of *Typhoid*, the relation being as 5 – 10 or 1 – 2, to 10 – 29 or slightly over 1 – 3. In the first collection its relative frequency in the two types was as 5 – 12 to 5 – 18. In this point of view the results of the two analyses show a pretty near correspondence. External otitis, leading to suppuration and purulent discharge from the ear occurred in *two Typhoid* cases.

*Involuntary Muscular Contractions.* As in the first collection of cases, the instances in which involuntary muscular contractions attracted notice, are very few. The facts, contained in the histories, are as follows:

Of the *Typhoid* cases, *which ended in recovery*, in *one*, moderate subsultus was observed on the *eleventh* and *fourteenth* days after admission; and in *one*, tremor of facial muscles when the patient spoke, on the *eighth* and *ninth* days. Of the cases of the same type *ending fatally*, in *one*, subsultus on the *second* day, and tremor of the upper extremities on the *fourth* day, which was the day before the death of the patient; in *one*, tremor of the upper extremities on the *twelfth* day.

Of the *Typhus* cases, *one* case, slight tremor of the body and limbs was observed on the *tenth* day. This case ended in recovery.

Of the cases of *Doubtful type*, in *one*, subsultus was observed on the *eighth* and *ninth* days. This case ended in recovery.

Tonic contraction of muscles, which occurred in a few of the cases analyzed by Louis, and also in those analyzed by Dr. Jackson, of Boston, was not noted in any of the cases included in this or the former collection.

*Carphologia.* This symptom is mentioned in the history of *two* fatal cases of *Typhoid*, in both of which muscular tremor of the extremities occurred. It is not stated to have occurred in any other cases.

Under the head of *prostration* I have nothing to add to what is said, on this point, in the first Report.

SECTION FIFTH.

*Symptoms referable to the Digestive System. Appetite, Thirst, Tongue, Sordes, Parotitis, Nausea, and Vomiting. Alvine dejections, Tympanites, Tenderness of Abdomen, Gurgling.* [No. for Oct. 1850, page 268.]

*Appetite.* Of the *forty-eight* cases, the histories are deficient in statements relative to the appetite in *ten.* As respects the remaining *thirty-eight* cases, the patients were asked if they desired, or relished food, and the information thus obtained, noted. In *twenty-seven* the patients declared they had no appetite, whenever asked, which was usually several times during the progress of the disease. Of course the stage of convalescence, and the period just preceding convalescence, are not now referred to, more or less return or appetite almost invariably signalizing a favorable change in the disease tending toward recovery. In *eleven* of the *thirty-eight* cases, the patients said that they had some desire for food, or that the food they received (which was essence of beef, and milk porridge) was acceptable.

With reference to these results some allowance is to be made for the uncertainty which, as has been seen, belongs to all the *subjective* phenomena of the career of Continued Fever. I do not feel sure that the patients always spoke the truth when they said they desired or relished food, but there is no way of disproving or confirming their statements. The results, to say the least, suffice to show that the idea of food does not affect the mind with disgust in all cases of fever. It is probable, indeed, that in a certain proportion of the cases, some appetite was felt. And, again, it is to be considered that the liability to error may apply to the cases in which patients declared they *had not*, as well as to the cases in which they affirmed that they *had* a disposition to take food. It is certain that patients affected with fever, after the disease has continued for some days, do not, in many instances, refuse nutriment, or take it with manifest reluctance. Sometimes, however, there is a strong repugnance to it. It is very rare that they ask for food, even although it may be readily taken when presented. The condition of the mind sufficiently accounts for the latter fact; and it is highly important to bear in mind, that, in so far as willingness to receive food, or even the existence of appetite, should have an influence on the propriety of administering nourishment, we must act without waiting for any spontaneous expressions on the part of the patient. The mind does not take that cognizance of the instinctive wants of the system which belongs to health. This is evidenced by various other particulars.

The presence of appetite is noted in a very much larger proportion of cases of the *Typhus* than of the *Typhoid* type. Excluding *three* cases in which nothing is noted on this point, of the *seven* remaining cases of *Typhus*, in *five* the patients said that they relished or desired food.

*Thirst.* In *ten* cases the histories do not state either the presence or absence of this symptom. Of the *thirty-eight* remaining cases, it existed to a greater or less extent. In *nineteen* of these cases the thirst was great during the early part of the febrile career. In a few cases it continued to be a prominent symptom throughout the disease, but generally, although intense at first, it lessened as the disease advanced. In some cases it was present only for the first few days. In only *one* case is it stated that there did not exist thirst at first, and in this case it became developed afterward. Information respecting this symptom, was obtained by questioning the patients, but frequently patients asked for drink, and complained of suffering from this source. This was true more especially at the early part of the disease, and, afterward, as I often noticed, although patients did not, of their own accord, indicate their desire for drink, if offered to them it was eagerly taken.

Thirst is, thus, a pretty constant symptom, more marked at the commencement of the febrile career, diminishing, or ceasing as the disease progresses, which, in part, at least, is probably in consequence of the bluntness of perceptions, the consciousness of the morbid condition upon which it depends being thereby impaired.

*Tongue.* In no instance did the tongue preserve a normal aspect throughout the disease. The morbid appearances were varied, not only in different cases, but at different periods in the same case. Without investigating the connections which these appearances had with other symptoms, with a view to determine, by that mode, the pathological significance and value which belong to them respectively, as was done in the former analysis, I will simply give enumerations of the cases in which the more prominent appearances were observed, bringing into comparison the different types, and the results in the two analyses. The appearances of the tongue were noted in all cases but one; that is in *forty-seven* cases.

*Dryness* of the superior surface of the organ, more or less in degree, extent, and duration, was noted in the histories of *twenty* cases of *Typhoid*, *eight* cases of *Typhus*, and *six* cases of *Doubtful type;* in all, *thirty-four*, of *forty-seven* cases. The first analysis gave this symptom in *thirty-six* of *fifty* cases. In both collections *seventy*, of *ninety-seven* cases.

There was notable resistance to the touch, or hardness of the superior surface, in *ten* cases of *Typhoid*, in *two* cases of *Typhus*, and in *two* cases of *Doubtful type;* in all, *fourteen*, of *forty-seven* cases. The first analysis give this appearance in *ten*, of *fifty* cases. In both collections, *twenty-four*, of *ninety-seven* cases.

The dryness was rarely observed at the early part of the febrile career, and was generally either confined to, or more marked on a central strip of the surface of the organ. It existed in every fatal case save one, and in the excepted case, the patient died suddenly, with apoplectic coma, the second day after coming under observation.

*Coating*, varying in thickness, color, and continuance, was present in *twenty-seven* cases of *Typhoid*, in *ten* cases of *Typhus*, and in *eight* cases of *Doubtful type;* in all, *forty-five*, of *forty-seven* cases. The first analysis gave *forty-seven* of *fifty* cases. In both collections, *ninety-two*, of *ninety-seven* cases. The color of the coating is not generally mentioned. It is stated to have been *dark* in *two* cases only, in both of which the disease ended favorably.

A *scabby* appearance was presented in *four* cases of *Typhoid*, in *one* case of *Typhus*, and in one case of *Doubtful type;* in all, *five*, of *forty-seven* cases. The first analysis gave *four*, of *fifty* cases. In both collections, *nine*, of *ninety-seven* cases. The surface exhibited fissures, or cracks, in *five* cases of *Typhoid*, in *one* case of *Typhus*, and *one* case of *Doubtful type;* in all, *seven*, of *forty-seven* cases. The first analysis gave *four*, of *fifty* cases. In both collections, *eleven*, of *ninety-seven* cases.

It had a very *smooth*, or *glazed* appearance in *three* cases of *Typhoid*, in *one* case of *Typhus*, and in no case of *Doubtful type;* in all, *four*, of *forty-seven* cases. The first analysis gave *five*, of *fifty* cases. In both collections *nine*, of *ninety-seven* cases.

A *reddened* aspect is noted in the history of *four* cases, all cases of *Typhoid*. The first analysis gave *eight* cases, all of the *Typhoid* type. In both collections, *twelve*, of *ninety-seven* cases.

Great difficulty in protruding the tongue existed in *three* cases of *Typhoid*, in *two* cases of *Typhus*, and in no case of *Doubtful type;* in all, *five* of *forty-seven* cases. The first analysis gave *six*, of *fifty* cases. In both collections, *eleven*, of *ninety-seven* cases. In each of the cases in this collection, in which great difficulty in protruding the tongue was noted, the disease proved fatal.

As respects any striking points of distinction between the two types, *Typhoid* and *Typhus*, these results are negative. The only feature which would appear, from my observations, to have any bearing on the diagnosis, is redness of the tongue. This was observed in a small proportion of the cases of *Typhoid* in both collections, and in not a single case of *Typhus*.

The comparison of the results in the two analyses, for the most part exhibits a similarity which is worthy of remark, as evidence that symptoms which, at first view, appear so uncertain, and, as it were, accidental, as the different appearances of the tongue, are, nevertheless, restricted in the frequency of their occurrence within certain numerical limits.

As already stated, the tongue frequently presented, in individual cases, during the progress of the disease, more or less of the various appearances that have been mentioned. The general facts with respect to this point, which were stated in the former Report, are equally applicable to the present collection of cases. In order, however, to illustrate the manner in which these appearances occur in succession in Continued Fever, I will give in a tabular form, the facts pertaining to the tongue as daily noted in the histories of *six* cases of either type, selecting one-half from the cases ending favorably, and the other half from those which were fatal.

In the following table the cases selected are placed in the separate columns numbered 1, 2, 3, etc., and the appearances noted on every day of the disease, in each case, are given in the column assigned to that case:

So far as the facts contained in the present collection of cases are developed, they go to confirm the correctness of the general conclusions submitted in the former Report, with respect to the importance of the tongue in furnishing indications in Continued Fever. The special attention bestowed, by some practitioners, on the various appearances which this organ assumes during the progress of the disease, and the significance attached to these appearances in the prognosis, and treatment, are, for the most part, based on notions purely speculative.

*Sordes.* The presence of this symptom is noted in the histories of *seventeen* of *forty-seven* cases in which the record of the phenomena pertaining to the digestive system is presumed to be complete. The first analysis gave *fourteen*, of *fifty-two* cases. In both collections, therefore, it was present in *thirty-one*, of *ninety-nine* cases.

The symptom was present in each of the fatal cases of *Typhus*, viz., in *four*, and in *four* of the *seven* fatal cases of *Typhoid;* in all, *eight*, of *eleven* cases. Thus, the proportion of cases in which it was observed that ended fatally, is as *eight* to *seventeen*, and, in the first collection, as *three* to *seven*.

Of the *seventeen* cases in which it is noted to have occurred, *ten* were cases of *Typhoid*, *six* were cases of *Typhus*, and in *one* the type was *Doubtful*. It was present, thus, in a larger ratio in the cases of *Typhus* than in those of *Typhoid*.

The periods in the disease, dating from the time the patients entered the hospital (which it is to be borne in mind was very rarely at the commencement of the disease), when sordes was first observed, in the cases respectively, are as follows:

| | |
|---|---|
| *Typhoid.* | |
| Second day, | Two cases. |
| Fourth day, | One case. |
| Fifth day, | Two cases. |
| Sixth day, | Three cases. |
| Fourteenth day, | One case. |
| Fifteenth day, | One case. |
| *Typhus.* | |
| First day, | One case. |
| Third day, | Two cases. |
| Eighth day, | Two cases. |
| Tenth day, | One case. |
| *Doubtful Type.* | |
| Fifth day, | One case. |

It is evident that sordes denotes a certain degree of gravity of disease, for it is not observed in mild cases, and is present in a proportion of the cases which prove fatal, considerably larger than in those which end in recovery. Nevertheless, it occurs not unfrequently in cases which recover, and is by no means present in all cases that are fatal. Hence, an unfavorable prognosis is not to be based on its presence, nor a favorable prognosis on its absence, although, in estimating the probabilities of the issue of the disease, the symptom is not without weight.

*Hæmorrhage from the gums.* This symptom is noted in the histories of *two* cases, the same number as in the first collection. In *one* case the disease was *Typhoid*, and in the *other* case the type was *Doubtful.* It occurred in the former case on the *fifth* day after the patient entered the hospital, and continued until the *eighth* day. The case became complicated with pneumonitis. The disease was not very severe, convalescence being pronounced on the *sixteenth* day after admission, and the *twenty-sixth* after the date of attack. In the latter case the hæmorrhage occurred on the *third* day after admission, and is only noted to have occurred on that day. Convalescence was pronounced in this case, on the *eighth* day after admission, and the *fourteenth* after the date of attack. In the first case petechiæ occurred on the abdomen and limbs, being intermingled, in the former situation, with the characteristic rose eruption. The petechiæ were true ecchymoses, not the maculæ of *Typhus.*

Inasmuch as in *three* of the *four* cases in which, in both collections, this symptom was observed, the disease terminated in recovery, it is to be inferred that its occurrence need not affect unfavorably the prognosis.

An *herpetic eruption about the mouth*, is noted in the histories of *three* cases, viz., *two* of *Typhus*, and *one* of *Typhoid.*

*Parotiditis.* This complication did not occur in any case. In this respect a comparison of the two collections exhibits a striking contrast, the first analysis having given *five* cases. These results go to show that parotiditis is not to be regarded as an intrinsic element of the disease, but one of the events which are due to certain special tendencies incident to the disease at particular times or places — tendencies, the nature and source of which are not susceptible of explanation with our present knowledge of the pathology of fever.

*Nausea and vomiting.* The occurrence of *vomiting* appears in the histories of *thirteen*, of *forty-seven* cases in which the record of the symptoms referable to the digestive system is presumed to be complete. Nausea, without vomiting, is noted in the histories of *four* cases. This proportion of cases is larger than in the first collection, in which vomiting occurred in *nine*, of

*fifty-two* cases. In the two collections this symptom occurred in *twenty-two*, of *ninety-nine* cases. In these enumerations the access, or forming stage of the disease is excluded, and, also, the period of the disease prior to the patient's coming under observation. With the exception of *one* case, it was not a prominent symptom, and, in the excepted case, it was not very prominent. In *four* of the cases in which it occurred the disease proved fatal. Of the *thirteen* cases, *eleven* were cases of *Typhoid*, *one* was a case of *Typhus*, and *one* a case of *Doubtful type*. Its relative occurrence was therefore greater in *Typhoid* than in *Typhus*. This was true, also, in the first collection.

The periods in the progress of the disease, at which the nausea or vomiting occurred, are as follows:

*Typhoid.*

Case 1. Vomited yellowish and greenish fluid day before death.
" 2. Vomited yellowish fluid on fourth day.
" 3. Nausea on the first and fourth days.
" 4. Vomited twice fourth day, and once seventh day.
" 5. Vomited once, after drinking freely of tea, on the fourth day.
" 6. Vomited once second, ninth, and tenth days; nausea eleventh and twelfth days; vomited thirteenth day. Rather a prominent symptom in this case.
" 7. Vomited first day.
" 8. Vomited fourth and fifth days.
" 9. Vomited seventh day, after taking porridge.
" 10. Nausea first day.
" 11. Vomited second day, after drinking.
" 12. Vomited first, second, third, and fifth days.
" 13. Nausea sixth day.
" 14. Vomited seventh day.

*Typhus.*

Case 1. Vomited fourth day.
" 2. Nausea fourth day.

*Doubtful Type.*

Case 1. Vomited twice during the first six days.

The days in the foregoing list are dated from the time the patients entered the hospital, not from the commencement of the disease.

The facts pertaining to this point show, that, during the febrile career, nausea and vomiting not only are absent in the majority of cases, but they are unimportant as symptoms, occurring at irregular periods, very seldom recurring, or persisting, and possessing no special significance.

*Alvine discharges. Typhoid. Diarrhœa*, more or less in degree, and duration, was present in *fourteen* of the the *twenty-nine Typhoid* cases. This result is as near as possible to that developed by the analysis of the hospital *Typhoid* cases in the first collection, which was *nine*, of *eighteen* cases. The two results could not approximate more closely without the division of a patient into halves!

The diarrhœa was mild, or slight in degree in all but *four* cases. This was the fact in the hospital *Typhoid* cases in the first collection, in all but *two* cases, the relation thus in both collections to each other being as 4 – 14, or 2 – 7, is to 2 – 9.

It was only present in the early part of the febrile career in *four* cases. This was the fact in the hospital *Typhoid* cases in the first collection in *three* cases, the relation in both collections thus being as 4 – 14 or 2 – 7, is to 3 – 9 or 1 – 3.

It was only present in the latter part of the career in *two* cases, one of which was fatal. This was the fact in the hospital *Typhoid* cases in the first collection in but *one* case; the relation thus being as 2 – 14 or 1 – 7, to 1 – 9.

It existed moderately through the febrile career in *six* cases. This was the fact in the hospital *Typhoid* cases in the first collection in *five* cases; the relation thus being as 6 – 14 or 3 – 7, is to 5 – 9.

Of the *seven* fatal *Typhoid* cases it was present in *three*. In the *four* hospital *Typhoid* cases in the first collection which proved fatal, it was present in *two;* the relation thus being as 2 – 4 or 1 – 2, is to 3 – 7.

*Typhus. Diarrhœa* was present in but *three* of the *ten* cases of *Typhus*. Of the *twelve* hospital cases in the first collection it was present in *four;* the relation thus being as 3 – 10, is to 4 – 12 or 1 – 3. In each of the three cases in which it was present, it was exceedingly mild in degree, and of short duration. In *one* of these cases it was only present on the day of admission, *four* dejections occurring on that day, and this may possibly have been the operation of a cathartic taken before entering the hospital. In another of the cases it consisted of *two* daily dejections on the second and third days after admission. In the remaining case it consisted of *two* daily dejections on the fifth, sixth, and seventh days after admission.

Each of these *three* cases of *Typhus* terminated fatally. Mild diarrhœa, therefore, existed in *three* of the *four* fatal cases of *Typhus*. Of the *two* fatal cases of *Typhus* in the first collection, diarrhœa was not present in either.

The term diarrhœa is used here in the same sense as before, viz., meaning too frequent, and liquid dejections.

It is also to be recollected, that these enumerations embrace only the career of the fever, exclusive of the access, or forming stage.

As respects this symptom, the two types, *Typhus* and *Typhoid*, exhibit a striking disparity; for, while in the *forty-seven* hospital *Typhoid* cases in both collections diarrhœa was present in *twenty-three*, or in the proportion of about *one-half*, in the *twenty-two* hospital cases of *Typhus* it existed in only *seven*, or in the proportion of about *one-third*. Moreover, it was universally very mild, or slight in the cases of *Typhus* in which it was present, while this was not true of all the cases of *Typhoid* into the histories of which it entered. A greater liability to the occurrence of diarrhœa, and its greater prominence as a symptom, are generally recognized as constituting one of the diagnostic features of *Typhoid* considered in contrast with *Typhus*, and it is to be remarked that in the grouping of the cases for these analyses more or less importance was attached to the presence or absence of this event.

*Constipation.* With reference to the facts pertaining to the alvine evacuations, it is particularly important to state that, with a very few exceptions, probably not more than three or four instances, no cathartic or laxative medicines were administered in the cases under analysis. The same statement will apply, nearly, if not quite as fully, to the hospital cases formerly analyzed. Consequently, the conditions of the bowels, as respects frequency of the dejections, and other symptoms, are such as belong to the disease uninfluenced by the remedies just mentioned. Enemas were occasionally employed, but these not often. Sometimes it happened that no dejections occurred for a longer period than would perhaps have been deemed advisable had the attention been more closely directed to this point, and pains always taken in individual cases, while they were in progress, to ascertain how many days had passed without an evacuation. The facts contained in the histories relative to constipation are not without interest and importance, and will doubtless strike the minds of some of my readers with surprise. I will give a synopsis of the histories in so far as the dejections are concerned, in several cases of the *Typhoid* and *Typhus* types, which were characterized by constipation.

*Typhoid.* *Case* 1. In this case the bowels remained quiescent for *five* days, and on the sixth day were moved by a simple enema. They were not moved for the *three* following days, when a single dejection occurred spontaneously. On the second day afterward, *three* or *four* dejections took place, and the following day *two;* the next day, none, and the next day, *one*, convalescence being then pronounced.

*Case* 2. In this case it is not stated whether the bowels were moved on the day of admission, or not; but no evacuation occurred subsequently for *six* days. On the eighth day after admission an evacuation occurred spontaneously; the ninth day, no dejection; on the tenth day, two dejections; on the eleventh and twelfth days, each, one dejection, the patient now being convalescent.

*Case* 3. In this case the bowels had not moved for *three* days prior to admission. They remained quiescent for *two* days after admission, and moved spontaneously on the *third* day, i. e., on the *sixth* day after the last preceding movement. On the day following, another dejection occurred, which was moulded, and perfectly natural in appearance — a phenomenon which is not likely to fall under the observation of practitioners who are accustomed to administer cathartics daily, or every other day, during the progress of the disease!

*Case* 4. In this case diarrhœa existed on the day of admission, several evacuations occurring on that day; afterward, no dejections occurred for *two* days, none are noted on the *third* day, none occurred on the *fourth* day, none are noted on the *fifth* day, and none occurred on the *sixth* day. On the *seventh* day an evacuation took place spontaneously. The next day, no dejection. The next day, one dejection. The next day, none. The next day, one. The two following days, none. The next day, two or three, and at this time convalescence was established.

*Case* 5. In this case one dejection occurred on the day of admission. Subsequently no dejection occurred for *six* days. On the *seventh* day there were two dejections, convalescence being at this time established.

It will be observed, that in each of the foregoing cases, the disease ended favorably, and, it should be added, that in none of the fatal cases was constipation present, with a single exception.

*Typhus.* *Case* 1. In this case, after the first day in hospital, on which there was one dejection, the bowels remained unmoved for *four* days. On the *fifth* day a stool occurred, the feces being moulded and natural; a single dejection occurred on each of the succeeding days, convalescence being pronounced on the following day.

*Case* 2. In this case no evacuation took place until the *fifth* day, when there were two dejections. On the two following days there was no dejection; on the next day none was noted; on each of the two following days, one dejection; on the next day one, and on the day following, two, convalescence being established.

*Case* 3. In this case no evacuation took place for the first *five* days in hospital. On the *sixth* day it is not noted whether the bowels were moved or not. On the *seventh* day there was no dejection. On the *eighth* day there was no dejection. In this case the patient was pronounced convalescent, but subsequently dysentery supervened, which proved fatal on the fifty-second day after admission. This is the only fatal case in which the bowels were permitted to remain unmoved for several days in succession.

I shall not deduce from the foregoing facts the conclusions that cases of fever are more likely to terminate favorably in which constipation exists, and that it is injudicious to interfere with this condition even if the bowels do not move for six, seven, or eight days. It is by no means claimed that the facts are sufficient to authorize these conclusions. It may, however, be fairly inferred that constipation is not generally attended with any unpleasant consequences, and does not appear to affect unfavorably the course of the disease. This inference would involve, as a corollary, that the practice of administering cathartics and laxatives in Continued Fever is not so necessary, or useful, as is by most practitioners supposed. Whether the practice be pernicious, or the extent to which it is so, must be settled by other observations. As a general maxim in therapeutics, it is certainly true that active remedies which are uncalled for, and useless, are, in proportion to their activity, hurtful; but I am not prepared to say that, in my opinion, this maxim is applicable to the point under consideration. Cathartics, or laxatives, may be required under certain conditions, when, in general, they may be needless, and even pernicious. It would be out of place to discuss this topic in the present connection. I will only add, that the practical rule pursued in the treatment of the cases under analysis was, not to prescribe remedies of this class so long as, on exploration of the abdomen, there appeared to be no evidences that the patient was suffering from undue retention of the excrement.

*Hæmorrhage from the Bowels.* This symptom is not noted to have occurred in any case. In the first collection it was present in *two* cases, both of the *Typhoid* type.

*Tympanites. Typhoid.* Tympanites, meteorism, or visible enlargement of the abdomen from an accumulation of gas, is noticed in the histories of *twenty-two* cases. The first analysis gave, of *eighteen* hospital *Typhoid* cases, this symptom in *twelve.* The relation in the two collections, therefore, is as 22 – 39 is to 12 – 18; and, in both collections, the symptom was present in *thirty-four,* of *forty-seven* cases. The tympanitic distension was *slight* in *five* cases, *moderate* in *thirteen,* and *considerable* in *four;* it would not be termed very great in degree in any case. These results accord with those developed by the first analysis.

*Typhus.* Of the *ten* cases of *Typhus* tympanites was present in *eight.* In the first collection, of *twelve* hospital cases, it existed in *eight.*

It was *slight* in every case but one, and in the excepted case was *considerable.* There was evidently a disparity in the *degree of tympanites,* in the cases belonging to the two types; in all the cases, save *one,* of *Typhus,* the symptom was less marked than in all the cases, save *five,* of *Typhoid* in which it was present. The first analysis showed the same disparity.

As respects the relative frequency of this symptom in the two types, in the first collection, the proportion of instances in which it was present *in the hospital cases,* was found to be exactly equal. The result, in the present collection, is not far from this, the proportion in the *Typhus* cases being larger by a small fraction. If the comparison be made with all the cases in the first collection, i. e., including the cases in private practice, with those in hospital, the results in both approximate still more closely, the *Typhus* cases in both having the symptom present in a slight fractional preponderance.

In the first Report I have expressed surprise at the fact of tympanites being found to be present in an equal ratio, or nearly so, in the two types. The symptom is considered somewhat distinctive of *Typhoid.* It is said to be very rare in *Typhus.* Entertaining this impression, I had thought that the facts contained in the first collection might have been affected by some peculiar circumstances, and that had the number of cases been much larger, the results would perhaps have been different. The similarity in the results of the two analyses, as respects this symptom, is thus worthy of particular note.

It is not to be overlooked that, in so far as my observations go, tympanites is less prominent as a symptom in the great majority of cases of *Typhus,* than in those of *Typhoid.* In this point of view the symptom furnishes a diagnostic feature of importance.

Examining the cases to ascertain the relation which this symptom sustains to *diarrhœa,* I find that of the *twenty-two* cases of *Typhoid* in which tympanites was present, diarrhœa co-existed in *six,* and was absent in *sixteen.* Diarrhœa consequently existed without tympanites in several instances. Of the *eight* cases of *Typhus* in which tympanites was present, diarrhœa co-existed in *three,* and was absent in *five.* The first analysis gave, of the *hospital Typhoid* cases, the presence of diarrhœa with tympanites in *eight,* and the existence of tympanites without diarrhœa in *four;* and of the *hospital Typhus* cases the co-existence of tympanites and diarrhœa in no case, in other words the absence of diarrhœa in every case in which tympanites was present. The results of the two analyses, as respects this point, are far from exhibiting uniformity, which goes the more to show that two symptoms, tympanites and diarrhœa, have little or no mutual dependence or connection.

With a view to the bearing of tympanites on the prognosis, it should not be omitted to state the proportion of cases in which it was present among those ending fatally. Of the *seven* fatal cases of *Typhoid* it was present in *six*, and in the excepted case the patient died the second day after admission with apoplectic coma. It existed in a *slight* degree in *one* case, it was *moderate* in *three*, and *considerable* in *two* cases. Of the *four* fatal *Typhus* cases, it was present in *three;* being *slight* in each. These results are precisely the same as in the first analysis. They show that the symptom occurs in a much larger proportion of the cases ending fatally, than of those in which recovery takes place. It is therefore, to a certain extent, a favorable indication when tympanites is not present.

*Abdominal tenderness.* More or less tenderness on pressure over the abdomen, was present during a part, or the whole of the febrile career, in *twenty-five* of the *twenty-nine Typhoid* cases, and in *five* of the *ten* cases of *Typhus.* As respects this symptom, thus, a point of contrast is afforded on comparison of the two types, it existing in by far the larger proportion of cases in *Typhoid.*

In degree it was either *slight* or *moderate* in all the *Typhoid* cases, excepting *two* in which peritonitis became developed after perforation of the intestine. It was *slight* in all the *Typhus* cases. It is evident from the histories that the degree of tenderness was less in all the cases of *Typhus* in which it was present, than in a large proportion of the cases of *Typhoid.* This symptom appears in the histories of the cases in this collection, in a larger ratio, in either type, than was developed by the first analysis. The latter gave it in *twelve*, of *eighteen hospital* cases of *Typhoid*, and, in *five*, of *twelve hospital* cases of *Typhus.* There is some room for error in determining the presence or absence of this symptom, as there is in all the *subjective* phenomena belonging to the history of Continued Fever. This may possibly account for the disparity in the results of the two analyses. The facts in both collections, however, authorize the deductions, which, for all practical purposes are sufficient, that more or less tenderness over the abdomen is a symptom belonging to both types of fever, but that it exists much oftener in *Typhoid* than *Typhus;* and that it is more or less prominent, as a symptom, in the former, while it is always slight in the latter.

The *tenderness* co-existed with *diarrhœa* in only *eight* cases of *Typhoid*, and in *two* cases of *Typhus.* This shows the absence of any relation between these symptoms.

It was associated with more or less meteorism in a large proportion of cases, viz., in *twenty* cases of *Typhoid*, and in *all* the cases (5) in which it was present in *Typhus*, i. e. in *twenty-five* of the *thirty* cases of both types in which tenderness existed. It would appear, therefore, that while the tenderness is not dependent on the presence of diarrhœa, it does sustain some connection with the meteoric distension of the abdomen. This would be the inference from the analysis of the present collection of cases; a different conclusion, however, was deduced from the results* of the first analysis. That analysis gave the co-existence of tympanites in but *ten* of *nineteen Typhoid* cases, and in *four* of *six Typhus* cases in which tenderness was present. This disparity in the results of the two analyses, and in the deduction based thereupon, shows the liability to error in numerical investigations from insufficient data. Expressed in figures, the relation of the two collections in this particular is as 14 – 25 to 25 – 30; the two symptoms thus co-existing in a proportion of a little over one-half in the first collection, and of more than two-thirds in the second. Adding together the cases in both collections, tenderness co-existed with tympanites in *thirty-nine*, of *fifty-five* cases. It is to be remarked, that the disparity of the results of the two analyses with respect to this point, is, in a measure, explicable by the fact that, in the present collection of cases, both tenderness and tympanites were found to be present in a larger relative number of cases than in the first collection. This would lead us to expect that the instances in which the two symptoms concurred would be more numerous. In conclusion,as regards the connection between the symptoms, the inferences from the facts enumerated in the first Report must be considered correct.

The situation in which the abdominal tenderness was more marked, or to which it was limited, is stated in the histories of all the cases, save two, in which the symptom was present. Of *twenty-four* cases of *Typhoid* it was limited to, or more marked in the *right iliac* region in *twenty*. It was limited to, or especially marked in *both iliac* regions in *two* cases. Of the *five Typhus* cases, the tenderness was limited to, or more marked in the *right iliac* region, in *three*.

---

* On reference to the portion of the former Report with a view to comparing the results, I find a typographical error in the figures, which, until now, had escaped notice. The error is obvious on comparing the summing up of the cases in which tenderness was present without tympanites, with the enumerations that have preceded. The reader is requested to correct the error in the first Report as follows: on page 333, line 19, *ten* should read *nine*, and the second *ten* should read *two*.

*Tenderness over the Epigastric* region was noted in the histories of *six* cases of *Typhoid*, and in *two* cases of *Typhus*. I have examined the histories of these cases to ascertain if, at the time the tenderness over the epigastrium was noted, there co-existed any gastric symptoms. The following are the facts with reference to this point: of the *six* cases of *Typhoid*, in *four*, *vomiting* appears among the symptoms recorded in connection with the epigastric tenderness; in *one*, *nausea*, without vomiting, co-existed; and in the remaining case, vomiting existed prior and subsequently to the tenderness being observed, but it was not noted in conjunction. Of the *two Typhus* cases, *vomiting* is not noted in the histories at any period of the febrile career. *Thirst* was present at the same time with tenderness in *four*, of the *six Typhoid* cases; in another case it had existed the day but one before the existence of tenderness is noted, and in the remaining case it was not present. In neither of the *two* cases of *Typhus* was *thirst* present.

*Perforation of Intestine.* This event occurred in one case, as was demonstrated after death, and was presumed, from the symptoms, to have occurred in another fatal case in which no autopsy was made. Both were cases of the *Typhoid* type. It did not occur in any of the cases before analyzed. Its numerical relation to the whole number of cases in both collections is, therefore, as *two* to *one hundred;* and to the whole number of cases of the *Typhoid* type, as *two* to *fifty-nine.*

In the first of the two cases mentioned, the perforation occurred on the sixteenth day after the admission of the patient. The fever had existed for several days before the patient entered the hospital. The disease was of about medium severity, and at the time of the perforation, the symptoms had exhibited improvement, and convalescence was apparently approaching. The case was complicated with pneumonitis.

On the morning of the day on which the perforation took place, the pulse was 108, and moderately developed. Skin warm and moist. She reported feeling comfortable. Cough diminished. Considerable tenderness of abdomen; two dejections, etc. At 12, noon, she was attacked with a severe chill, followed by heat and perspiration. She complained of acute pain in abdomen. The pulse, seven hours afterward, was 132, small, and feeble. Tenderness of abdomen extreme, and general. Considerable tympanites.

On the following day the symptoms were as follows: Expression of anxiety. Respirations 28, inspiration short and quick. Hands cold, and of a livid hue. Pulse inappreciable. Sordes on teeth. Tongue dry and hard. One copious dejection. Abdomen much distended and tympanitic, and tender on pressure. Replies to questions slowly, dozing when not aroused, with eyeballs upturned. Died in the evening of this day, twenty hours after the perforation occurred.

In the case in which perforation is presumed to have occurred, the patient was a female, fourteen years of age. The disease in this case, was severe, and complicated with pneumonitis. The occurrence of perforation was denoted by the development of severe pain in abdomen, great meteorism, extreme tenderness, prostration, the pulse rising from 118 to 132, being very small and feeble, etc. The precise time when the accident was supposed to have occurred, is not stated. Death took place in somewhat less than forty-eight hours afterward. An autopsy was not permitted in this case.

*Gurgling.* The presence of this symptom is frequently mentioned, but pains were not taken to note its presence or absence with sufficient uniformity to render an analysis with respect to it of much value.

(*To be continued.*)

